# The effect of sense of security on job performance of medical staff: the mediating effect of psychological capital

**DOI:** 10.3389/fpsyg.2024.1347783

**Published:** 2024-04-04

**Authors:** Yixuan Xu, Ningjing Zhan, Dantong Zhang, Zhanghao Xie, Gege Li, Huigen Huang

**Affiliations:** ^1^Department of Nursing, Guangdong Provincial People's Hospital (Guangdong Academy of Medical Sciences), Southern Medical University, Guangzhou, China; ^2^Department of Nursing, Shantou University Medical College, Shantou, China; ^3^Department of Nursing, Guangdong Pharmaceutical University, Guangzhou, China; ^4^Department of Nursing, Jinan University, Guangzhou, China; ^5^Department of Nursing, Southern Medical University, Guangzhou, China

**Keywords:** medical personnel, job performance, sense of security, psychological capital, mediating effect

## Abstract

**Objective:**

To investigate the current situation of sense of security, psychological capital and job performance of medical staff in Guangdong Province, and to explore the mediating role of psychological capital on the relationship between sense of security and job performance of medical staff.

**Methods:**

In this study, 969 health care workers were selected from February 2023 to April 2023 from 37 hospitals in Guangdong Province, China, using purposive sampling method. The Sense of Security Scale for Medical Staff (SSS-MS), psychological capital scale (PCS) in Chinese version and the Chinese version of job performance scale (JPS) were used in this study. We use SPSS 26.0 for statistical analysis and Amos 24.0 for structural equation modeling (SEM). The control variables entering SEM were selected by regression analysis. SEM analysis confirmed psychological capital scale's mediating function in the link between work performance scale and Sense of Security.

**Results:**

The overall SSS-MS, PCS, and JPS scores were 67.42 ± 16.136, 87.06 ± 15.04, and 77.87 ± 10.50, respectively. The results of Pearson's correlation analysis showed that there was a positive relationship between PCS and JPS (*r* = 0.722, *P* < 0.01), SSS-MS and JPS (*r* = 0.312, *P* < 0.01), and SSS-MS and PCS (*r* = 0.424, *P* < 0.01). PCS demonstrated a fully mediating influence on the link between medical workers' SSS-MS and JPS, according to structural equation modeling.

**Conclusion:**

The JPS of medical personnel in Guangdong Province is at a medium level, with much room for improvement. PCS is positively impacted by a sense of security. There is a supportive correlation between PCS, JPS, and SSS-MS. Furthermore, PCS fully mediates the relationship between medical staff members' JPS and their SSS-MS. The Job Diamond-Resource model and Conservation of Resource theory are further validated and supplemented by the findings of this study, which also gives managers a theoretical foundation for enhancing medical staff performance.

## 1 Introduction

Security is defined as a confident, stable, carefree and calm emotional state, especially the feeling that current and future needs can be met (Ba et al., 2021). A sense of security can be regarded as an indicator of mental health status. Safety in the workplace has received more attention in recent years, particularly in healthcare settings. The research pointed out that the SSS-MS is not optimistic, only about 24.55% of medical personnel feel safe at work (Gui et al., 2013). Violent attacks against medical personnel are also widespread worldwide (Rathert et al., 2018). At the same time, Chinese medical personnel are faced with a more prominent risk of hospital violence, and the proportion of violence against medical personnel is as high as 42.2%−83.3% (Sun et al., 2017; Li et al., 2018). In addition, the heavy workload of hospitals, internal competition and other pressures will lead to the lack of security of medical staff, which will further affect the mental health and work efficiency of healthcare providers, and ultimately affect the medical quality of hospitals (Ilczak et al., 2021). As a result, extensive efforts to increase healthcare staff security are required, with the goal of ensuring the quality of medical care as well as professional personnel rights and interests.

With the implementation of the “14th Five-Year Plan,” the National Health Commission requires efforts to improve medical workers' service quality and pointed out that Medical services concentrate on the quality of medical care and nursing (Cheng et al., 2021). The work performance of medical personnel is the achievements and contributions made in medical activities, and the behavior performance as well as results which related to their medical work (Scotter and Motowidlo, 1996). High level of work performance is the key to enhance the quality of medical care. However, there are diverse factors that affect the performance of medical staff, among which psychological factors play a great role (Tong, 2018). Burke et al. (2015) pointed out that nurses' low sense of security produced negative work attitude, decreasing job satisfaction, affecting work performance and improved nursing service quality. He Y. et al. (2023) pointed out that security can effectively improve doctors' work involvement, further increase work effort and work performance, and improve work performance. Social exchange theory (Wang Z. et al., 2021) points out that if one party in a social interaction does something beneficial to the other party, the other party has the obligation to return the favor to maintain the exchange relationship. Based on the reciprocity principle in the social exchange theory, when the organization shows support, openness and trust, medical staff will feel that the organization gives them enough protection and security, think that they enjoy more autonomy, get more resources, and feel more respect from the organization. In exchange, they will return in a certain manner and attempt to enhance their work performance under the direction of the business. Relevant studies have also confirmed this view (Wang W. et al., 2023). It is evident that the sense of security of medical personnel affects work performance.

Positive psychological states that a person exhibits during their process of personal development are referred to as psychological capital. Positive psychological states are those in which a person responds proactively to life's dilemmas, such as optimism, who keeps one's confidence undiminished and guides one to deal with the challenges of reality in a more stable way. It is a significant characteristic of individual variability that has drawn increasing attention from scholars, including self-efficacy, hope, resilience and optimism (Luthans et al., 2004). Studies have suggested that among various factors such as doctor-patient relationship perception, job satisfaction and job involvement, psychological capital has the greatest influence job performance, and the development of psychological capital helps medical staff improve job performance (Wu et al., 2019). According to the JD-R, all job features fall into either of the two categories: requirements or resources. Job resources are a category of physiological related resources that are beneficial in promoting the achievement of employees' work goals, including income, organizational support, etc. In the JD-R model, the gain path states that abundant work resources stimulate employees to generate higher work engagement, which in turn results in beneficial outcomes like strong organizational commitment and strong retention intentions (Patel and Bartholomew, 2021). Psychological capital can be seen as an important predictive factor of security and job resource. Psychological capital is a personal resource. When people get supported by organizations, friends and family, they have a more positive attitude and they feel more secure. When a individual has a substantial degree of psychological capital, the sense of security is further enhanced (Chiesa et al., 2018). The above speculation leads to the conclusion that security and psychological capital can stimulate medical personnel to produce higher job performance through the gain path. Previous researches have pointed that security is positively correlated with work performance (Pourteimour et al., 2021). Studies have indicated a tight relationship between JPS and PCS (Zhao et al., 2021). Meanwhile, the sense of security is also an important psychological capital predictor (Jiang and Zhou, 2020).

**Figure 1:**
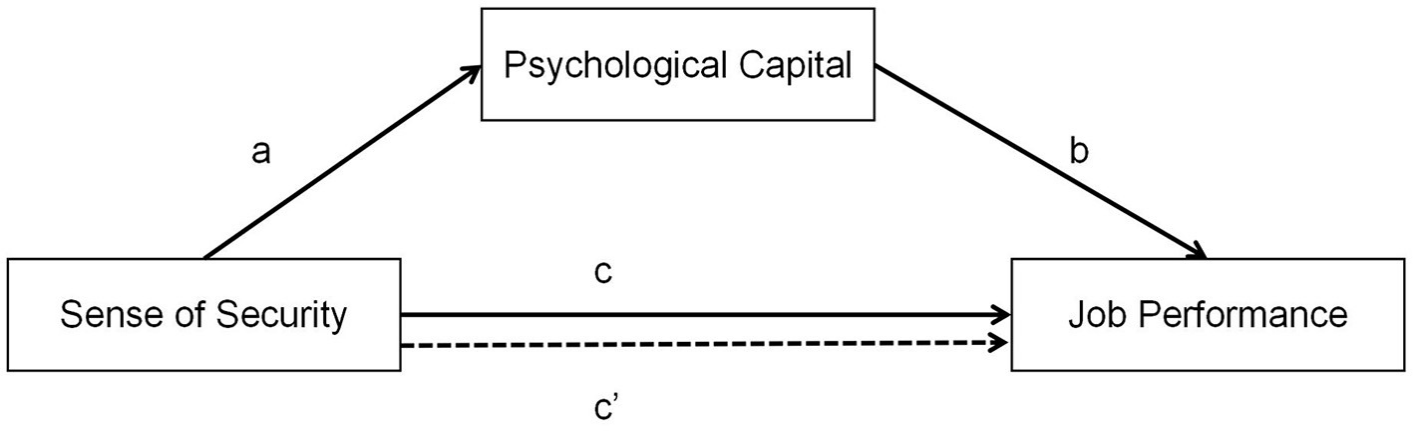
The theoretical model.

To sum up, we hypothesize a strong relationship between medical staff members' psychological capital, sense of security, and work performance. At present, the relationship between these three has not yet been supported by relevant literature, in which the influence mechanism has not been explored, and the current domestic research on the above mentioned mostly focuses on the fields of corporate employees, family relations, educational psychology, etc., and is less focused on the medical field. Using the JD-R model as a basis, we suggest the following model conceptual diagram based on the aforementioned information, as shown in Figure 1.

This research aim to deeply explore the impact mechanism of medical staff's sense of security and psychological capital on job performance, in order to give a theoretical foundation for managers to understand the psychological needs of medical staff, manage their human resources, promoting excellence health care, and facilitating the implementation of quality health services measures.

We propose the following hypotheses:

H1: SSS-MS is positively correlated to JPS (path a).
H2: SSS-MS is positively correlated to PCS (path c).
H3: PCS is positively correlated to JPS (path b).
H4: PCS mediates the relationship between SSS-MS and JPS (path c′).

## 2 Materials and methods

### 2.1 Study design and participants

From February to April 2023, a purposive sampling method was used for selecting healthcare professionals in Guangdong Province as the research objects. The following requirements have to be fulfilled by participants: (a) medical staff with a license or registration and (b) at least 1 year work experience in the hospitals. Hospital workers who were unwilling to participate in the study or suffering from mental illness were excluded. For 80% power, a sample of 290 providers was required with an alpha of 0.05 (Maccallum et al., 1996; Chuanji et al., 2020). A number >348 is required in the final sample in order to account for a 20% failure to respond rate. There were 1,057 questionnaires distributed in all, and 969 (91.67%) of them were properly returned. The Guangdong Provincial People's Hospital Medical Ethics Committee has authorized this study [approval number: KY2023-480-02], and all study participants have provided their informed consent.

### 2.2 Data collection

A web-based questionnaire platform called “SurveyStar,” developed by Changsha Ranxing Science and Technology, Shanghai, China, was used to conduct the survey. It was designed for avoiding respondents from being questioned beyond once. An email inviting administrators at hospitals to take part in the research was sent to them. They were requested to forward it to the team as soon as they consented to participate in the research. The purpose of the study was explained to participants, and they were assured that the information they provided would remain confidential. Once they are willing to take part in the research, informed consent was acquired. Only individuals who agree take part and provided complete answers to the questionnaire were eligible to submit it.

### 2.3 Measurements

#### 2.3.1 Sense of security scale for medical staff

The sense of security scale for medical staff (SSS-MS) developed in the early stage by our research group (Ba et al., 2021) was adopted, including 22 items in five dimensions including environment, organization, self, patients, social support and management. We used the likert 5-point scoring method where all items were inversely rated from strongly agree to strongly disagree is shown by the numbers 1 through 5. The sum of all criteria, which ranged from 22 to 110, was the final score. The degree of sense of security increases with score. This scale's Cronbach's alpha in this investigation was 0.952.

#### 2.3.2 Psychological capital scale

The Psychological capital scale (PCS) was developed by Luthans and its validity and reliability of Chinese version were confirmed by Luo Hong (Luthans et al., 2004). The scale contains 20 items in four dimensions: resilience, hope, self-efficacy and optimism. It adopts Likert 6-level scoring method, and 20 items are scored positively. A range from strongly disagree to strongly agree is shown by the numbers 1 through 6. The total score range from 20 to 120 points. Higher psychological capital is indicated by a higher score. The scale's Cronbach's alpha in this research was 0.962.

#### 2.3.3 Job performance scale

The job performance scale (JPS), developed by Motowidlo and Scotte (Motowidlo and Van Scotter, 1994), contains 16 items in three dimensions: job dedication, task performance and interpersonal promotion. Likert 6-level scoring method is adopted, with 1 indicating “strongly disagree” to 6 indicating “strongly agree.” Higher scores indicate greater work performance. The overall score might vary from 16 to 96 points. The job performance questionnaire is applied in China with high validity and reliability; its Cronbach's α coefficient is 0.956 for total internal consistency.

#### 2.3.4 Participants' demographic characteristics

Age, gender, work experience (in years), job position, job title (primary medical title, Intermediate medical title or Senior medical title), educational level (master's or doctor's degree; bachelor's degree; junior college; technical secondary school), marital status (separated or divorced; single; married), monthly income, overtime per week, sleep satisfaction, work satisfaction and operational field were the demographic characteristics collected. The population for this research was split up into eight age groups: 18–25, 26–30, 31–35, 36–40, 41–45, 46–50, 51–55 and >55 years. The positions include nurses, doctors, technicians, and pharmacists. Nurses are responsible for providing care to patients who are ill or injured, while doctors treat patients. Technicians help diagnose disease while pharmacists produce and distribute medications for patients. Five monthly income categories were created out of the participants: <5,000 yuan, 5,001–10,000 yuan, 10,001–15,000 yuan, 15,001–20,000 yuan, >20,000 yuan. The participants were divided into six overtime per week groups: 0–5 h per week, 6–10 h per week, 11–15 h per week, 16–20 h per week, 21–25 h per week, >25 h per week. Sleep satisfaction and work satisfaction were graded into five levels: Very dissatisfied, dissatisfied, intermediate, satisfied, very satisfied, respectively.

### 2.4 Statistical analyses

AMOS version 24.0 (IBM, Armonk, NY, USA) and SPSS Statistics version 26.0 (SPSS Inc., Chicago, IL, USA) were used for the statistical analysis. With SPSS, descriptive statistics, regression analysis, and Pearson's correlation were employed. Initially, percentage and frequency were used to describe data with a regularly distributed distribution for categorical variables, while mean and standard deviation were used for continuous variables. Second, the association between PCS, JPS, and SSS-MS was examined using Pearson correlation analysis. SEM is a method used to test and specify linear relationship models between observed and latent variables. The control variables were included in the structural equation model after regression analysis of demographic variables (Shrout and Bolger, 2002).

Through the use of relative and absolute indices, SEM evaluated the model's goodness-of-fit index using the maximum likelihood estimation approach. Among these were chi-square, chi-square degrees of freedom (χ^2^/df), Tucker-Lewis index (TLI), incremental fit index (IFI), comparative fit index (CFI), normative fit index (NFI), root mean square error of approximation (RMSEA), and goodness-of-fit index (AGFI). The values that followed were considered to be acceptable: χ^2^/df <5, TLI > 0.90, RMSEA ≤ 0.08, AGFI > 0.90, GFI > 0.90, IFI > 0.90, CFI > 0.90, and NFI > 0.90 (Kang and Ahn, 2021).

To create the model, the SSS-MS, PCS, and JPS underwent confirmatory factor analysis in the first step. The internal reliability was assessed using Cronbach's α coefficient and composite structure reliability, and the validity of the measuring instrument was examined using confirmatory factor analysis. We next used the statistical program AMOS version 24.0 to do a mediation study in order to evaluate our hypothesis. To find its mediating influence, we determined SSS-MS to be the independent variable, JPS to be the dependent variable, and PCS to be the mediator variable in order to determine its mediating impact (Figure 1).

### 2.5 Ethical considerations

The Guangdong Provincial People's Hospital Ethics Committee gave its approval to this study (KY2023-480-02). Participants signed a written consent agreement before to implementation, which made it apparent that the questionnaire would be filled out anonymously. The researchers kept the data they collected to ensure privacy and avoid any unintentional usage.

## 3 Result

### 3.1 Descriptive statistics

The sample included 969 medical staff (nurses, doctors, technicians and pharmacists) from 36 tertiary hospitals dispersed over Guangdong Province's four areas (the Pearl River Delta, western, northern, eastern). Among the 969 healthcare workers, 225 (23.22%) were doctors, 622 (64.19%) were nurses, 40 (4.13%) were pharmacists, and 82 (8.46%) were technicians, with 209 (21.57%) men and 760 (78.43%) women. In terms of education, 62 (6.4%) possessed a technical secondary school degree, 175 (18.06%) possessed a junior college degree, 630 (65.02%) possessed a bachelor's degree, 80 (8.26%) possessed a master's degree and 22 (2.27%) possessed a doctor's degree. Table 1 displays the remaining demographic features of this sample.

**Table 1:** Sociodemographic characteristics of medical staff (*N* = 969).

**Variables**	**Categories**	***N***	**%**
Gender	Men	209	21.57%
	Women	760	78.43%
Age	18–25	83	8.57%
	26–30	261	26.93%
	31–35	180	18.58%
	36–40	123	12.69%
	41–45	129	13.31%
	46–50	85	8.77%
	51–55	79	8.15%
	>55	29	2.99%
Work experience (in years)	1–5	200	20.64%
	6–10	255	26.32%
	11–15	151	15.58%
	16–20	109	11.25%
	21–25	90	9.29%
	26–30	164	16.92%
Job position	Doctor	225	23.22%
	Nurse	622	64.19%
	Pharmacist	40	4.13%
	Technician	82	8.46%
Job title	Primary medical title	497	51.29%
	Intermediate medical title	292	30.13%
	Senior medical title	180	18.58%
Educational level	Doctor's degree	22	2.27%
	Master's degree	80	8.26%
	Bachelor's degree	630	65.02%
	Junior college	175	18.06%
	Technical secondary school	62	6.40%
Marital status	Married	702	72.45%
	Single	248	25.59%
	Divorced/separated	19	1.96%
Monthly income (yuan)	<5,000	186	19.20%
	5,001–10,000	440	45.41%
	10,001–15,000	183	18.89%
	15,001–20,000	73	7.53%
	>20,000	87	8.98%
Overtime per week (hours per week)	0–5	388	40.04%
	6–10	387	39.94%
	11–15	99	10.22%
	16–20	48	4.95%
	21–25	14	1.44%
	>25	33	3.41%
Sleep satisfaction	Very dissatisfied	95	9.80%
	Dissatisfied	232	23.94%
	Intermediate	416	42.93%
	Satisfied	203	20.95%
	Very satisfied	23	2.37%
Operational field	Eastern Guangdong	504	52.01%
	Western Guangdong	112	11.56%
	Northern Guangdong	17	1.75%
	The pearl river delta	336	34.67%
Job satisfaction	Very dissatisfied	27	2.79%
	Dissatisfied	75	7.74%
	Intermediate	410	42.31%
	Satisfied	388	40.04%
	Very satisfied	69	7.12%

### 3.2 Model identification

When the standardized factor loadings (between 0 and 1) of the measurement model have higher values, it indicates that the observed variables are more indicative of the latent variables. To reflect the reliability and validity of each construct, in this study we used three measurements specific to SEM: average variance extract (AVE), composite reliability (CR) and factor loading. Table 2 indicated all the constructs's CRs were >0.85, suggesting that the entries were adequate indicators. In addition, the AVE values demonstrated that each latent variable had discriminant validity and sufficient convergent based on the CFA results.

**Table 2:** Reliability and validity test of the measurement model (*N* = 969).

**Scale**	**Dimension**	***M* ±SD**	**Factor loading**	**Cronbach's alpha**	**AVE**	**CR**
Sense of security scale for medical staff	Environment	11.32 ± 3.24	0.740	0.768	0.616	0.889
	Patients	11.57 ± 3.57	0.754	0.925		
	Self	10.10 ± 2.49	0.723	0.846		
	Organizational management	22.37 ± 5.98	0.882	0.888		
	Social support	12.06 ± 3.88	0.815	0.919		
Psychological capital scale	Self-efficacy	26.66 ± 4.73	0.864	0.896	0.777	0.933
	Hope	25.62 ± 5.01	0.885	0.914		
	Resilience	21.83 ± 3.99	0.922	0.903		
	Optimism	12.95 ± 3.01	0.854	0.936		
Job performance scale	Job dedication	27.80 ± 4.28	0.937	0.878	0.694	0.871
	Task performance	24.47 ± 3.69	0.792	0.933		
	Interpersonal promotion	25.61 ± 3.64	0.759	0.955		

### 3.3 Relationships between healthcare workers' sense of security, psychological capital, and job performance

As presented in Table 3, SSS-MS was positively correlated with PCSl (*r* range from 0.331 to 0.466, *P* < 0.01) and JPS (*r* range from 0.242 to 0.322, *P* < 0.01). PCS was also positively correlated with JPS (*r* range from 0.575 to 0.731, *P* < 0.01; Table 3). Hypotheses 1–3 were proven to be valid, which also suggests that these three variables can be included in structural equation modeling for analysis.

**Table 3:** Correlations among observed indicators in the SEM (N = 969).

	**1**	**2**	**3**	**4**	**5**	**6**	**7**	**8**	**9**	**10**	**11**	**12**	**13**	**14**	**15**
1	–														
2	0.563^**^	–													
3	0.546^**^	0.601^**^	–												
4	0.643^**^	0.577^**^	0.644^**^	–											
5	0.608^**^	0.625^**^	0.545^**^	0.767^**^	–										
6	0.794^**^	0.792^**^	0.767^**^	0.911^**^	0.869^**^	–									
7	0.281^**^	0.191^**^	0.371^**^	0.333^**^	0.213^**^	0.331^**^	–								
8	0.350^**^	0.224^**^	0.413^**^	0.405^**^	0.287^**^	0.403^**^	0.744^**^	–							
9	0.296^**^	0.228^**^	0.403^**^	0.358^**^	0.248^**^	0.365^**^	0.691^**^	0.817^**^	–						
10	0.371^**^	0.277^**^	0.407^**^	0.437^**^	0.354^**^	0.446^**^	0.628^**^	0.763^**^	0.781^**^	–					
11	0.358^**^	0.250^**^	0.443^**^	0.422^**^	0.300^**^	0.424^**^	0.871^**^	0.937^**^	0.911^**^	0.859^**^	–				
12	0.263^**^	0.171^**^	0.344^**^	0.340^**^	0.217^**^	0.322^**^	0.646^**^	0.634^**^	0.712^**^	0.637^**^	0.731^**^	–			
13	0.207^**^	0.129^**^	0.302^**^	0.238^**^	0.155^**^	0.242^**^	0.582^**^	0.572^**^	0.611^**^	0.520^**^	0.640^**^	0.737^**^	–		
14	0.205^**^	0.135^**^	0.290^**^	0.317^**^	0.177^**^	0.276^**^	0.531^**^	0.506^**^	0.534^**^	0.487^**^	0.575^**^	0.717^**^	0.730^**^	–	
15	0.251^**^	0.161^**^	0.346^**^	0.331^**^	0.204^**^	0.312^**^	0.651^**^	0.635^**^	0.690^**^	0.611^**^	0.722^**^	0.915^**^	0.904^**^	0.895^**^	–

1, Environment; 2, Patients; 3, Self; 4, Organizational management; 5, Social support; 6, Sense of security scale for medical staff; 7, Self-efficacy; 8, Hope; 9, Resilience; 10, Optimism; 11, Psychological capital Scale; 12, Job dedication; 13, Task performance; 14, Interpersonal promotion; 15, Job performance scale.

^**^P <0.01 (two-sided).

### 3.4 Mediation analysis between medical professionals' sense of security, psychological capital, and job performance

**Figure 2:**
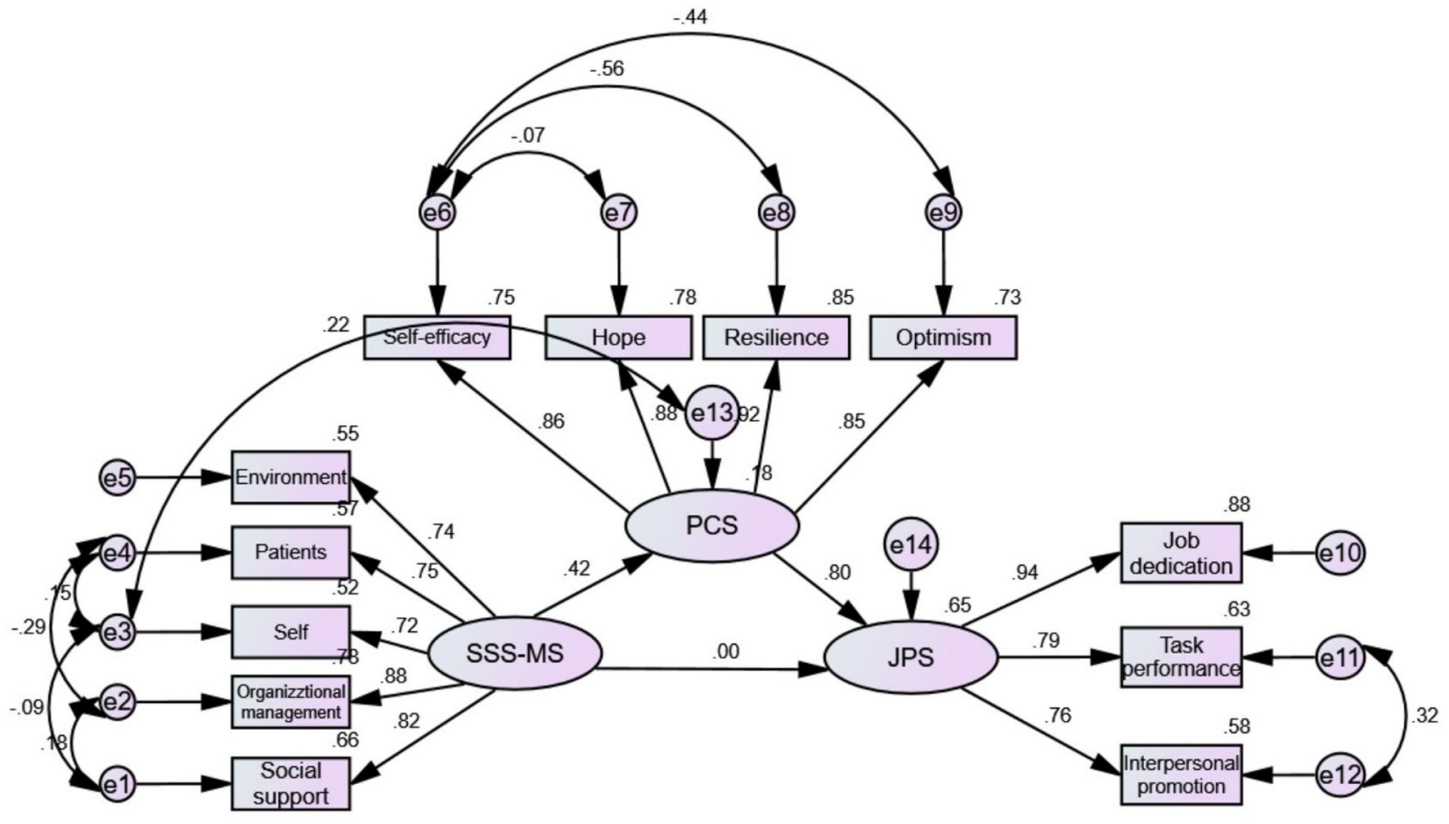
Structural equation model of sense of security, psychological capital, and job performance (standardized).

The model explains 42.5% of the total security variance. Figure 2 displays the final model's standardized path coefficients, and Table 4 presents the model's standardized and unstandardized path coefficients. Regression analysis examined how work performance was impacted by demographic traits.

**Table 4:** Unstandardized and standardized path coefficients for the structural model.

	***B***	**β**	**S.E**.	**C.R**.	***P***
SSS-MS → PCS	0.550	0.425	0.046	12.031	^***^
PCS → JPS	0.785	0.803	0.037	21.188	^***^
SSS-MS → JPS	0.004	0.003	0.035	0.113	0.910

^***^P < 0.001 (two-sided).

Among the demographic characteristics, only work satisfaction (β = 2.688, *P* < 0.001) and job title (β = 2.954, *P* < 0.001) show a significant effect on the job performance, which were subsequently incorporated into the final model as control variables. According to the model fit, CMIN/DF = 4.849 (<5), RMSEA = 0.063 (<0.080), all absolute and relative index values were >0.90.

As seen in Figure 2, SSS-MS-MS was positive correlated to PCS (β = 0.425, *P* < 0.001), PCS was positively correlated to JPS (β = 0.803, *P* < 0.001), but SSS-MS-MS was uncorrelated to JPS (β = 0.003, *P* = 0.91). Additionally, PCS plays an indirect protective role on JPS while affecting the SSS-MS (β = 0.425 * 0.803 = 0.341, *P* < 0.001). The findings indicated that PCS completely mediated the association between SSS-MS and JPS. It means that H4 is proved to be valid.

## 4 Discussion

On the basis of theory, a hypothesis model was constructed to investigate the connection between SSS-MS and JPS in this study, and lead psychological capital as an intermediary variable. Our findings confirm our hypotheses. The findings demonstrated that SSS-MS is positively independent affecting PCS, and PCS is positively correlate to JPS. Furthermore, SSS-MS positively affects JPS. In addition, the link between SSS-MS and JPS is mediated by PCS. Further analysis showed that different dimensions of SSS-MS (self, patients, social support, environment and organizational management) were all positively correlated with job performance. This means that each of these components can serve as a facilitator of job performance. Meanwhile, psychological capital regulates the relationship between these factors to some extent. The results may provide more justification for managers to improve the performance of medical staff from the psychological field. Therefore, managers should show more concerned about the mental health status of healthcare personnel, and actively manage and develop their psychological capital. In summary, this research has significant theoretical and practical implications for improving work performance and management level of medical personnel.

### 4.1 Current status of medical staff's job performance

In this study, JPS of medical staff was (77.87 ± 10.50), which indicated that the overall job performance of medical staff was above the average level. This score is close to the results of Xu Fan's study on medical staff in 4 public tertiary hospitals in Sichuan, Zhejiang and Fujian provinces (Xu et al., 2023). The average scores of the dimensions showed that dedication was the highest and task performance was the lowest. The highest score in the dimension of job dedication may be attributed to the fact that the medical staff in this study are younger, mostly with a bachelor's degree or above, and are in a critical period of striving, so they are more willing to try challenging jobs higher than their own level (Li et al., 2022). At the same time, the sense of accomplishment and responsibility brought by the profession of medical personnel may also motivate them to increase work engagement, resulting in better performance (Levin et al., 2022). The score of interpersonal promotion dimension is above the middle level, suggesting that medical personnel have harmonious relationships with their coworkers. This may result from the large proportion of medical personnel with moderate or above job satisfaction in this study. High job satisfaction contributes to positive emotions and vitality, providing a sense of psychological security and comfort and producing positive behavioral outcomes (Susanto et al., 2022). Low task performance may be associated with a fast-paced, high-intensity work environment, which leads to energy and enthusiasm depletion (Liu et al., 2022), affecting motivation and engagement and reducing task performance (Guo et al., 2021). In turn, lower work quality can exacerbate doctor-patient conflicts, further deepening the work pressure of medical staff, forming a vicious cycle and adversely affecting task performance (Wang Z. et al., 2023). Based on the above analysis, it is recommended that managers adjust the allocation of human resources, reduce energy consumption, actively respond to emergencies, and improve work performance.

### 4.2 The correlation of sense of security, psychological capital and job performance among medical personnel

The results of the study showed that medical staff's sense of security positively affects psychological capital. The sense of security provides a solid foundation for psychological capital. When medical staff feel safe at work from the security provided by patients, environment, organizational support, etc., it promotes the growth of psychological capital. They will face the challenges of life with a positive and optimistic attitude, believing that they are capable of coping with difficulties and full of confidence in the future, and this kind of confidence and hope is an important part of psychological capital. According to the findings of this study each dimension of PCS was positively related with JPS, which is the same as other studies (Wang et al., 2022). This indicated that medical staff who maintained high psychological capital would have better performance in their work. As a crucial element of positive psychological resources, PCS has an important impact on individual attitude, behavior and JPS (Brunetto et al., 2016). Falco et al. (2018) pointed out that the inner positive psychological state show a positive guiding impact on individuals, enabling individuals to show stronger stress resistance and resilience in the face of difficulties, cope with negative emotions at work with an optimistic attitude, improve their adaptability to their working environment, and help better balance life and work, thus improving work efficiency. The study of Chen and Liu (2022) also found that when nurses had a higher level of psychological capital, they were better able to complete basic work, master professional skills, abide by hospital rules and regulations, and maintain a good relationship with colleagues. These positive psychological states and characteristics make them perform well in their work, and their work performance is improved accordingly. Therefore, managers should show more concerned about the mental health status of Medical professionals and enhance their work performance by effectively managing and developing their psychological capital.

### 4.3 The mediating effect of psychological capital on the relationship between sense of security and job performance among medical professionals

The SSS-MS in the workplace show a direct positive effect on their JPS, which is not only directly related to JPS of medical professionals, but also has an indirect positive impact through PCS. Security refers to the basic needs that medical staff feel subjectively in their work. Only when they feel safe can they experience inner satisfaction and get more benefits from their work (Ayalew and Workineh, 2019). The concept consists of five interrelated components that interact and collectively influence the sense of security of medical personnel. As an important part of medical staff's work resources, security is of great significance to their mental health and career development. When medical staff feel insecure, this bad perception will be transformed into work pressure (He Q. et al., 2023), which may lead a negative effect on the working mood and motivation of medical staff, resulting in the negative emotion of job burnout, loss of confidence in clinical work. Furthermore, it also reduces their psychological capital, which ultimately leads to low work performance. According to the theory of resource conservation (Wang S. et al., 2021), individual psychological capital is the core psychological element to improve work performance and promote personal development. High psychological capital medical personnel are more capable and have superior resources, and can take various ways to cope with emergencies or emergencies in clinical work, and also have strong willpower and confidence to complete clinical tasks (Xu et al., 2020). This positive attitude is conducive to cultivating their clinical learning and working ability and improving their core competence. As an intermediary, psychological capital, regulates the association between medical staff's work performance to some extent. This process reveals the power of positivity, further confirming the JD-R model (Patel and Bartholomew, 2021). Therefore, medical personnel with a higher sense of security will have more confidence and motivation to work, obtain more material and spiritual resources from all aspects, and constantly improve their work performance.

This study verifies the relationship between SSS-MS, PCS and JPS, and the mediating effect of PCS in the interaction between SSS-MS and JPS. This study aim to improve the security and psychological capital of healthcare workers, so as to improve the job performance of medical staff. To achieve this goal, it's essential for the health system, hospital administrators and all social parties to work together to strengthen the investment in the medical field, so as to ensure and promote the stable and sustainable development of the medical team. Furthermore, the results of this study further validate and supplement the relevant contents of the COR theory and JD-R model (Hobfoll, 1989). In the Chinese context, it is of special significance to study the role of PCS in the relationship between security and JPS. Treating SSS-MS and PCS as working resource, and emphasizing how psychological capital functions as a link between available resources and work performance, will help medical staff to improve work performance. At the same time, medical institutions should fully realize the significance of providing sufficient working resources for medical staff. By establishing a good sense of security environment to promote mutual trust within the organization, while training and improving the psychological capital of medical staff, it can create more motivation for work resources, so as to more actively commit to the cause of the organization and raise the attachment degree to the organization. This point of view provides a new perspective for improving work performance.

## 5 Conclusion

To sum up, different age, occupation, department, length of service, number of night shifts per month, employment relationship, marital status, monthly income, job satisfaction, sleep condition, number of workplace violence and work region have differences on the job performance of medical practitioners in Guangdong Province. SSS-MS is positively related to both PCS and JPS. PCS was also positively related to JPS. PCS has a comprehensive mediating effect between SSS-MS and JPS. There is room for improvement in their work performance, and the stability of their talent team needs to be strengthened. Improving the sense of security and psychological capital of healthcare professionals contribute to improving their working motivation, and then improve their work performance. Relevant departments should show concern about the healthcare professionals' psychological capital, and constantly enhance the mental health of healthcare professionals, so that they can better provide residents with more complete, comprehensive and personalized services.

There are some limitations in this study. Firstly, since it is a cross-sectional study, we merely examine the association among psychological capital, work performance, and security; we were incapable to determine a cause-and-effect link among these variables. In order to make up for this deficiency, future longitudinal studies need to be further confirmed. Secondly, due to the application of purposive sampling, the findings of the research may be limited in universality. To enhance the representativeness of the sample, we selected 36 hospitals distributed in the Pearl River Delta of Guangdong Province, Eastern, Western and Northern of Guangdong Province, and selected based on the proportion of hospitals in each of the four areas. Finally, due to cost and temporal limitations, every measurement was self-reported. Despite the risks of over-reporting and under-reporting, all of the scales that are utilized have strong psychometric qualities and are commonly employed.

Regardless of these shortcomings, this study provided theoretical and practical terms to earlier research. On the theoretical side, this study expands the application scenarios of the job requirement-resource model and explores mediation models that contribute to further understanding of the relationship among SSS-MS, PCS and JPS. In practice, these findings are crucial for improving the intrinsic resources and job performance of healthcare workers. They also have far-reaching significance for managers to continuously improve medical services and solidly promote the high-quality development of health care.

## Data availability statement

The original contributions presented in the study are included in the article/supplementary material, further inquiries can be directed to the corresponding author.

## Ethics statement

The studies involving humans were approved by Guangdong Provincial People's Hospital Ethics Committee from Guangdong Provincial People's Hospital (Guangdong Academy of Medical Sciences). The studies were conducted in accordance with the local legislation and institutional requirements. The participants provided their written informed consent to participate in this study.

## Author contributions

YX: Formal analysis, Writing – original draft, Writing – review & editing. NZ: Investigation, Writing – original draft. DZ: Investigation, Writing – original draft. ZX: Data curation, Writing – review & editing. GL: Data curation, Investigation, Writing – review & editing. HH: Project administration, Writing – review & editing.
